# A Case of Tension Pneumomediastinum Treated With Mediastinal Drainage Using a Semi-flexible Fiberscope via a Subxiphoid Approach

**DOI:** 10.7759/cureus.56599

**Published:** 2024-03-20

**Authors:** Yuji Matsumoto, Hiroshi Mizuuchi, Kokoro Honjo, Masahiro Hata, Takehiko Shigenaga

**Affiliations:** 1 Respiratory Medicine, Oita Redcross Hospital, Oita-shi, JPN; 2 Respiratory Surgery, Oita Red Cross Hospital, Oita-shi, JPN; 3 Respiratory Medicine, Oita Red Cross Hospital, Oita-shi, JPN

**Keywords:** a subxiphoid approach, idiopathic pulmonary fibrosis (ipf), decompression of the mediastinum, mediastinal drainage, semi-flexible fiberscope, tension pneumomediastinum

## Abstract

Tension pneumomediastinum with hemodynamic failure is a rare but life-threatening condition. Rapid decompression of the mediastinum by drainage is essential to save the patient's life. This report presents a case of tension pneumomediastinum that developed during conservative management of a pneumomediastinum associated with idiopathic pulmonary fibrosis. Endoscopically guided mediastinal drainage was successfully performed in the emergency situation of tension pneumomediastinum. Using the semi-flexible fiberscope inserted through a subxiphoid approach, the drainage catheter was easily and safely placed at the appropriate site in the mediastinum. Good mediastinal decompression was achieved, and the patient was out of this critical condition.

## Introduction

Pneumomediastinum is a rare occurrence in patients with idiopathic pulmonary fibrosis (IPF). However, it is associated with an unfavorable outcome and can be considered a potential predictor of mortality in IPF [1]. In this report, we present a case of a patient with IPF who developed tension pneumomediastinum, a potentially fatal condition caused by an increase in intra-mediastinal pressure leading to cardiac filling restriction and reduced cardiac output known as extracardiac obstructive shock [2,3]. Hemodynamic failure progresses so rapidly that without timely diagnosis and therapeutic intervention; it becomes irreversible and leads to death. In situations requiring urgent mediastinal decompression, such as tension pneumomediastinum, there are no established guidelines for rapid, safe, and effective removal of air from the mediastinum. In practice, the management of such situations depends on the skill of the attending physicians. This report demonstrates mediastinal drainage using the advantages of the semi-flexible fiberscope.

## Case presentation

A 78-year-old man with IPF, who has been receiving pirfenidone 1800 mg for two years, presented to our department with anterior chest pain and respiratory distress following exertion. Computed tomography (CT) revealed honeycombing in both lung fields and air collection in the mediastinum (pneumomediastinum or mediastinal emphysema). Once he was treated for presumed clinical pneumonia, 16th day after admission for observation, he developed a fever of 38.0℃, and his cough worsened. Laboratory data showed a white blood cell count of 30,500/µL and a C-reactive protein level of 38.3 mg/dL. The CT scan showed consolidation in the lower lobe of the right lung, which is indicative of pneumonia. Additionally, there was extensive mediastinal emphysema from the neck to the diaphragm. Sputum culture showed Klebsiella aerogenes and bacteria resident in the oral cavity. Antibiotic therapy using tazobactam/piperacillin 13.5g/day was initiated to treat presumed clinical pneumonia. However, the patient experienced severe hypotension and tachycardia with a blood pressure of 62/42 mmHg and a pulse of 140 bpm the following day. Despite high-volume infusions, hemodynamic instability did not improve, and continuous intravenous noradrenaline injection of 0.2γ was required. The patient was diagnosed with tension pneumomediastinum with extracardiac obstructive shock caused by massive mediastinal emphysema. Urgent mediastinal decompression by drainage was necessary.

Emergency mediastinal drainage was performed as follows: A 2 cm skin incision was made in the subxiphoid area under local anesthesia. After blunt dissection with a pean forceps toward the posterior surface of the sternum, a port was inserted. A semi-flexible fiberscope (EVIS LUCERA Thoracic Video Scope OLYMPUS LTF TYPE 260, OLYMPUS, Germany) was then inserted into the mediastinum through the port, and its position was confirmed fluoroscopically. The endoscopic forceps were used to dissect connective tissue and create an acceptable space for appropriate drain placement. Care was taken not to damage the mediastinal pleura and pericardium. While observing the interior of the mediastinum with the fiberscope, a silicone drainage catheter (6.5mm multichannel thoracic drainage catheter) was inserted through the port site and positioned appropriately (see Video 1). The catheter was connected to a negative-pressure water seal drainage bag for drainage of the mediastinum.

**Video 1:** The process of reaching into the mediastinum and placing the drainage catheter using endoscopic and forceps techniques.

**Figure 1:**
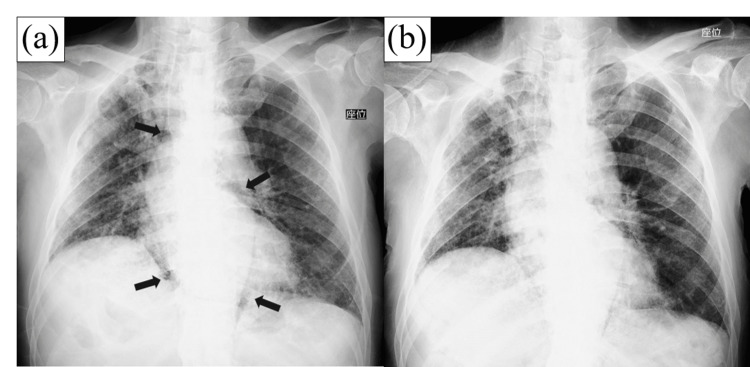
(a) Chest X-ray during mediastinal emphysema exacerbation (arrows). (b) Chest X-ray after mediastinal drain insertion. The drain was placed in the mediastinum, and emphysema subsequently resolved.

The patient's vital signs gradually improved after drainage with a blood pressure of 105/64 mmHg and a pulse of 100 bpm. On the 20th day, noradrenaline was ended with a gradual decrease by the POD 3rd day, and the patient's symptoms of shortness of breath and chest tightness resolved. Supplemental oxygen was no longer required, and a follow-up chest CT showed a decrease in mediastinal air accumulation (see Figure 1 and Video 2). The drain was removed on the 30th day after confirmation of no recurrence of a pneumomediastinum. There were no significant complications during or after the procedure. The patient later died due to terminal respiratory failure resulting from IPF progression on the POD 35th day. No recurrence of a pneumomediastinum was observed until death.

**Video 2:** Chest CT scan before (left) and after (right) mediastinal drainage. Left: Extensive mediastinal emphysema is seen from the neck to the diaphragm. Right: Significant reduction of air accumulation in the mediastinum is shown. CT: Computed tomography

## Discussion

Pneumomediastinum is typically caused by air leakage into the pulmonary interstitium resulting from alveolar rupture. The free air in the pulmonary interstitium migrates through the peribronchovascular interstitium toward the mediastinum. This pathophysiologic process is known as the Macklin effect [4]. It can explain the pathogenesis of pneumomediastinum and can be associated with bronchial asthma [5], interstitial pneumonia [6], SARS-CoV-2 infection [7], barotrauma in mechanical ventilation [8], and blunt chest trauma [9]. In patients with PF, rupture of the alveoli or honeycomb cysts and subsequent air leakage into the surrounding interstitium could be regarded as the cause of pneumomediastinum [10]. Pneumomediastinum can also be caused by injury to the trachea, central bronchi, esophagus, or peritoneal cavity resulting from iatrogenic, traumatic, or nontraumatic factors [2,11,12]. 

Conservative therapy involves avoiding known triggers and resting [2]. However, in rare cases, excessive air accumulation in the mediastinal space can increase pressure on vital cardiovascular and respiratory systems, leading to cardiovascular collapse, namely extracardiac obstructive shock. This condition is known as tension pneumomediastinum and requires immediate mediastinal decompression to reverse extracardiac obstructive shock [2,3]. Since the coronavirus disease 2019 (COVID-19) epidemic, there have been increasing reports of tension pneumomediastinum in COVID-19 patients, especially those receiving intubated ventilation [13-16]. Other reported cases of tension pneumomediastinum include those due to esophageal rupture after severe vomiting [11], tracheal perforation during cardiopulmonary resuscitation [12], and interstitial pneumonia [6].

Classically reported invasive surgical techniques for tension pneumomediastinum include tracheostomy, sternotomy, and mediastinotomy, all of which carry a risk of complications such as bleeding, hemothorax, damage to adjacent structures, and infection. In recent years, many reports have demonstrated the effectiveness of percutaneous mediastinal tube drainage under CT guidance [17-20]. This method can be superior to invasive surgical techniques in that it is quicker, safer, and less invasive. On the other hand, various drain insertion approaches have been proposed for mediastinal drainage, including suprasternal, parasternal, subxiphoid, and anterior chest wall, but no consensus has emerged as to which one is superior. In practice, the choice of drain insertion route for tension pneumomediastinum is based on CT imaging, targeting the largest air reservoir in the mediastinum and selecting a safe route that does not damage blood vessels or the lung parenchyma.

To the best of our knowledge, endoscopy-guided percutaneous mediastinal drainage has not been reported. In this case, we used a semi-flexible fiberscope designed for thoracoscopy, which has a rigid tube and is easy to maneuver in the mediastinum. It was inserted through the port placed in the subxiphoid area, which allowed easy access to the mediastinum. The endoscopic forceps were useful in dissecting connective tissue to create space for proper drain placement. The flexible tip of the fiberscope allows good visualization in narrow mediastinal spaces, even in obese patients with significant adipose tissue in the mediastinum. The drainage catheter was placed under the guidance of endoscopic visualization of the internal structures of the mediastinum. Despite the patient's vital signs indicating shock, the procedure was performed quickly and safely without complications such as bleeding or organ damage and may be feasible for mediastinal decompression in emergencies such as tension pneumomediastinum.

## Conclusions

Tension pneumomediastinum is a rare but fatal condition requiring immediate mediastinal drainage. The use of a semi-flexible fiberscope with endoscopic forceps was shown to be beneficial for drain placement. Endoscopic visualization of the mediastinum during the procedure makes it safer and easier than conventional blind indwelling drain placement. Therefore, endoscopically guided drain placement may be a viable option for mediastinal drainage in emergency situations such as tension pneumomediastinum.
